# Supplementary material for: Effect of Potassium Doping on the Structural and Catalytic Properties of Co/MnOx Catalyst in the Steam Reforming of Ethanol
Source: Materials (Basel). 2023 Jul 31;16(15):5377. doi: 10.3390/ma16155377 (PMC10420196; doi:10.3390/ma16155377)
Supplement: Supplementary file 1 [file materials-16-05377-s001.zip › materials-2133406-supplementary.pdf]

Supporting Information

Effect of Potassium Doping on the Structural and Catalytic Properties of Co/MnO_x Catalyst in the Steam Reforming of Ethanol

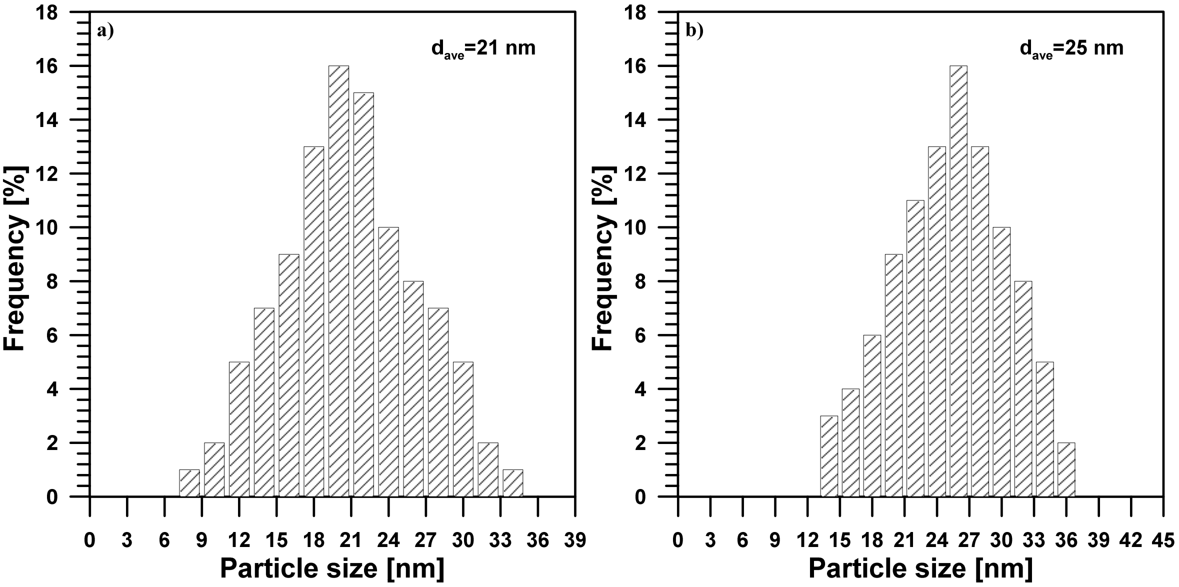

Figure S1. Particle size distribution histograms for (**a**) Co/MnO_x and (**b**) KCo/MnO_x catalysts reduced at 500 °C.
